# Synthesis and properties of novel styrene acrylonitrile/polypropylene blends with enhanced toughness

**DOI:** 10.1186/s13065-018-0447-9

**Published:** 2018-07-09

**Authors:** Yi-jun Liao, Xiao-li Wu, Lin Zhu, Tao Yi

**Affiliations:** 1 School of Materials Engineering, Chengdu Technological University, Chengdu, 611730 China; 2 School of Chinese Medicine, Hong Kong Baptist University, Hong Kong, Special Administrative Region People’s Republic of China

**Keywords:** Polypropylene, Styrene acrylonitrile, Nanoparticles, Toughness

## Abstract

**Background:**

Although polypropylene (PP) has been widely used, its brittleness restricts even further applications.

**Methods:**

In this study, we have used a melt blending process to synthesize styrene acrylonitrile (SAN)/PP blends containing 0, 5, 10, 15 and 20 wt% SAN. The effects of adding various amount of SAN on the blends characteristics, mechanical properties, thermal behavior and morphology were investigated.

**Results:**

The results demonstrated that SAN had no obviously effect on crystal form but reduced the crystallinity of PP and increased the viscosity. The heat deflection temperature and Vicat softening temperature were enhanced for all SAN/PP blends, in particular for blends with low SAN content (5 and 10 wt%). The morphology of SAN/PP blends with 10 wt% SAN revealed the presence of nanoparticles dispersed on the surface, while SAN/PP blends with 20 wt% SAN exhibited the presence of spherical droplets and dark holes. All SAN/PP blends showed higher impact strength compared to pure PP, especially for SAN/PP blend containing 10 wt% SAN for which the impact strength was 2.3 times higher than that of pure PP.

**Conclusions:**

The reason for significant increase in impact properties seemed to have a strong correlation with nanoparticles morphology and the decrease of PP crystallinity.

## Background

Thermoplastic polymers have been extensively used in our life duo to their advantages of recyclability, sustainability and superior properties [1]. Polypropylene (PP) is one of thermoplastic polymers, which has attracted considerable attention in the past decades owing to its outstanding mechanical properties, easy formation, excellent electrical insulation, high resistance to chemical agents, and environmental friendliness [2, 3]. While PP has a variety of serious defects, such as large molding shrinkage, low notch impact resistance at low temperature, especially low resistance in crack propagation despite its high resistance to crack initiation [4, 5]. Nowadays, the method of improving the toughness of polymers is mainly adding modifiers such as a plastic, an elastomer or a rigid body [6–9]. The combination of rubber or a thermoplastic elastomer with a polymer is one of the most effective toughening modifications; however, as the content of modifier increases, the elastic modulus, tensile strength and high-temperature creep deformation of the composites are significantly reduced [10, 11]. In recent years, researches have been experimenting with adding rigid bodies to polymer blends to improve impact strength. The rigid bodies not only toughen the blends, but also enhance their overall physical properties for specific applications [12–15]. Adding organic rigid bodies to PP is a common method for increasing the impact resistance of PP, with appropriate modification of the interface [16, 17]. Use of the organic rigid bodies nylon-6 [18, 19], polymethyl methacrylate [20, 21] and acrylonitrile–butadiene–styrene (ABS) [22–25] has been frequently reported in recent years. Mai et al. [14] synthesized nine groups of polypropylene blends with different organic rigid bodies, demonstrating that polycarbonate/polymethyl methacrylate (PC/PMMA) could improve the impact strength of the PP matrix. Bonda et al. [16] synthesized ABS/PP blends with compatibilizers and demonstrated that the increase of impact strength was due to the rubber toughening effect of ABS. In contrast, blending the organic rigid body styrene acrylonitrile (SAN) with PP has been much less frequently reported.

SAN resins are copolymers of styrene (PS) and acrylonitrile (AN). ABS is a terpolymer of acrylonitrile, butadiene and styrene in which styrene provides rigidity and ease of processability, acrylonitrile supplies chemical resistance, rigidity and heat stability, and butadiene (PB) supplies toughness and impact strength [26, 27]. SAN, without PB, is brittle and has low impact strength, and is expected to be an organic rigid body that can enhance the impact strength of PP like inorganic particles [22]. There are two theories of reinforcement polymers matrix for inorganic particles, one is that adding inorganic rigid particles may cause changes in the distribution of the stress concentration in the polymer and yielding strength in some area under low stress, and finally result in the enhancement effect on impact strength of polymer. Another theory is that rigid particles resist the crack propagation of the polymer matrix, followed by making it being passivated and ultimately prevent the fissure developing into destructive cracking in the process of plastic deformation [28–30]. Adding an organic rigid body like SAN to PP may be better for impact strength than adding inorganic particles because SAN can bond with PP due to the presence of acrylonitrile [31].

Nevertheless, different from inorganic particles, the compatibility between organic particles and the polymer matrix needs to be well controlled, which would significantly affect the diameter of dispersed particles and adhesion strength (the morphology), thus causing possible changes in mechanical properties. Kim et al. [32] controlled the morphology and interfacial tension of PC/SAN blends with a compatibilizer, indicating that PC/SAN blends had minimum interaction energy as adding PC to SAN polymer. Kum et al. [33] examined the influence of PP-g-SAN on a PP/ABS system, and obtained minimum droplet size at an optimized compatibilizer ratio and enhanced the interaction between both phases, and thus subsequently affected the mechanical and morphological properties. Several scientific works have stated that the incompatibility of PP and the ABS matrix arises from huge differences in their polarity and thermal coefficients. Therefore, study of the compatible effects of SAN and PP matrix is necessary in order to systematically examine the effect of SAN on the mechanical and thermal properties of PP matrix.

Compared with ABS, SAN shows lower impact strength due to lacking butadiene, which is similar to the more rigid inorganic particles. Besides, it is easy to control the compatibility and chemical bonds with PP. However, blending styrene acrylonitrile (SAN) with PP have been much less frequently reported. Herein, in this study, we focus on comparing mechanical performances, the morphology, and thermal deformation properties of SAN/PP blends obtained by a melt-blending process using a twin-screw extruder. The contents of SAN were selected at 0, 5, 10, 15 and 20 wt% because these were expected to enhance toughness and optimize thermal deformation properties of the blends.

## Methods

### Materials

Polypropylene (PP, MFI = 27 g/10 min) was purchased from Kingfa Science and Technology. Co., Ltd. SAN (HF-1095A) was purchased from Huafeng Corporation (Guangzhou, China). Chlorinated paraffin (CP) was obtained from Shanghai Sunny New Technology Development (Shanghai, China). Styrene maleic anhydride (SMA) bought from Shanghai Sunny New Technology Development (Shanghai, China). The starting compositions of the respective blends are presented in Table 1. All materials used in the blends were first dried at 80 °C and then accurately weighed.

**Table 1:** The composition of pure PP and SAN/PP blends

Blends	PP (wt%)	SAN (wt%)	SMA (wt%)	CP (wt%)
SAN/PP-0	97.5	0	2	0.5
SAN/PP-5	92.5	5	2	0.5
SAN/PP-10	87.5	10	2	0.5
SAN/PP-15	82.5	15	2	0.5
SAN/PP-20	77.5	20	2	0.5

### Synthesis of SAN/PP blends

The SAN/PP blends were prepared by melt-blending process with slight modifications [23, 34]. Initially, SAN, PP, SMA, and CP were pre-blended in a high speed mixer (SHR-10A, Coperion Heng AO Machinery, Nanjing, China). Then the mixtures were melted and blended using a twin screw co-rotating extruder (SHJ-36, Coperion Heng AO Machinery, Nanjing, China) with L/D 40 operating at a speed of 30 rpm/min. Compounding was carried out at 165, 175, 180, 185, 190, 195 and 190 °C in sequential heating zones was cooled, cut, and then dried at 90 °C for 8 h to remove all the water before characterization. Some extrudate was immediately molded in an injection molding machine (TC-150-P, Tiancheng Machinery Co. Ltd, China) at 180, 195, and 205 °C in sequential zones from hopper to mold to obtain dog-bone shaped sheets of 150 mm × 10 mm × 4 mm and rectangular samples of 80 mm × 10 mm × 4 mm for mechanical (tensile, impact tests), thermal (heat deflection and Vicat softening temperatures, melt flow index test and morphological examination (scanning electron microscopy).

### Characterization

The phase constituents of five blends were evaluated using an X-ray diffractometer (XRD, Philips PC-APD) with a CuKα (30 mA and 30 kV) radiation source of 0.154 nm wavelength at room temperature of 25 °C. The functional groups were examined using a Fourier transform infrared spectroscope (FTIR, Nicolet, 170SX, Wisconsin, USA) in the wave number range of 400–4000 cm^−1^ by pressing the samples and KBr into a membrane. The thermal properties of the blends were determined using a differential scanning calorimeter; samples were subjected to a stream of pure nitrogen flowing at a rate of 50 ml/min and heated at 10 °C/min from 25 to 220 °C.

The degree of crystallinity (X_c_) of PP was determined by calculating the ratio of heat of fusion (△H_m_) of the specimens to the heat of fusion of 100% crystalline PP (△H_m_ = 207 J/g) [35].

### Mechanical properties testing

Measurements of the tensile strength and elongation at break of all specimens were carried out on a universal testing machine (WDW-100, Tianjin Meites Testing machine factory, China) using dog bone-shaped specimens (150 mm × 10 mm × 4 mm) according to the standard of GB/T 1040.2-2006 at room temperature. The assay was performed under a liner deformation loading rate of 50 mm/min until mechanical failure occurred. Three replicates were performed for each measurement.

The impact strength was assessed on a beam impact testing machine (XJJ-5, Chengde Shipeng Testing Machine Co. LTD, China) at ambient temperature using rectangular samples (80 mm × 10 mm × 4 mm) in terms of GB/T 1043.1-2008 standard. For each measurement, three specimens were used.

### Morphological observations

The morphologies of PP and SAN/PP blends containing 10 or 20 wt% SAN were characterized by scanning electron microscopy (SEM, S-900, Hitachi) at magnifications of 2000X and 10,000X, operating at an accelerating voltage of 5 kV. The specimens were cryogenically fractured in liquid nitrogen, and the fracture surfaces were coated with platinum to a depth of 10 Å.

### Thermal deformation behavior and viscosity analysis

The melt flow indexes (MFI) of PP and SAN/PP blends were determined using a flow rate meter (XNR-400B, Chengde Shipeng Testing Machine co. LTD, China) using particle specimens at 230 °C with a loading weight of 2.16 kg in accordance with GB/T 3682-2000 standard.

The thermal deformation properties of PP and SAN/PP blends were assessed using a thermal deformation and Vicat softening temperature tester (XWB-300B, Chengde Shipeng Testing Machine co. LTD, China) with silicone oil as warming medium. Rectangular samples (80 mm × 10 mm × 4 mm) were scanned from 25 °C to deformation temperature at a heating rate of 120 °C/h under a perpendicular loading weight of 75 g (bending normal stress: 0.45 MPa) in line with GB/T1634.2-2004. The Vicat softening temperatures of the specimens were measured under a loading weight of 1000 g, heating from 25 °C to Vicat softening temperature at a rate of 50 °C/h in terms of GB/T 1633-2000.

## Results and discussion

### XRD studies of SAN/PP blends

**Fig. 1:**
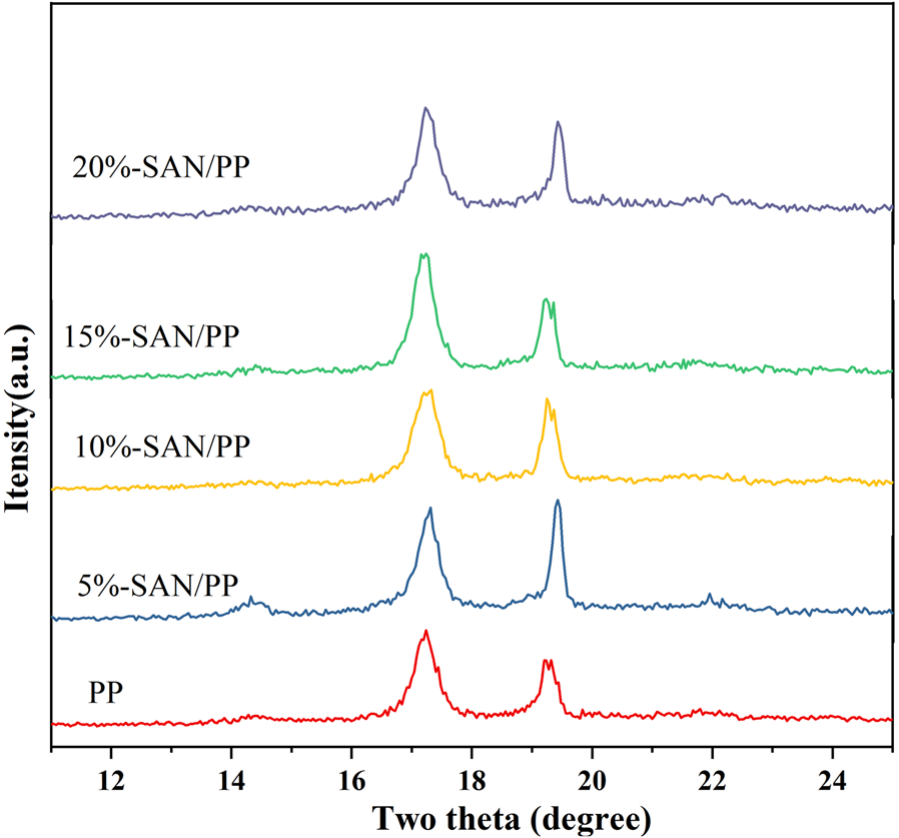
XRD patterns of PP and SAN/PP blends

It is known that PP is a polymorphous crystal, showing three crystalline forms designated as α-phase, β-phase, and γ-phase. α-phase is the dominanting; β-phase and γ-phase are induced when nucleating agents are added into the PP matrix [23–25]. The XRD patterns of pure PP and SAN/PP blends are displayed in Fig. 1. Crystal peaks can be clearly observed at 2θ values of around 14.2°, 17.1°, 19.2° and 21.7° for all specimens. These peaks were consistent with the monoclinic α-form of PP crystals for (110), (040), (130) and (131) planes, respectively [36]. However, the peaks of β and γ-crystalline phases did not occur, and there were no significant difference for all specimens; these results indicate that SAN has no obvious effect on crystallization behavior of PP.

### FTIR analysis of SAN/PP blends

**Fig. 2:**
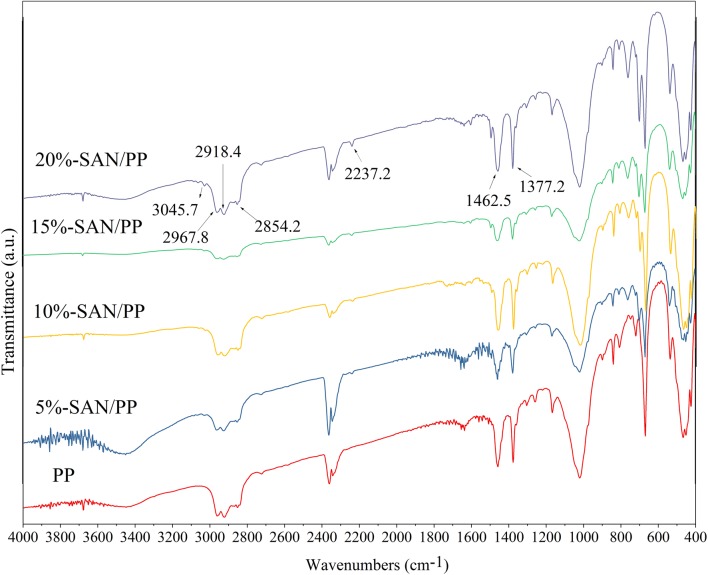
FTIR spectra of PP and SAN/PP blends

Figure 2 shows the FTIR spectra of pure PP and SAN/PP blends. The characteristic peaks of PP were observed for all specimens. The absorption peaks of 2967.8 and 2918.4 cm^−1^ are consistent with symmetric and asymmetric stretching vibrations of CH_2_ or CH_3_, and the peak at 2854.2 cm^−1^ corresponds to symmetric and asymmetric vibrations of CH_3_ [37, 38]. In addition, the peak around 1462.5 and 1377.2 cm^−1^ was assigned as the CH_3_ or CH_2_ deformation vibration. In contrast to pure PP, the FTIR spectrum of SAN/PP blends was clearly different, exhibiting two additional peaks around 2237.2 and 3045.7 cm^−1^ that correspond to C–N stretching vibrations in acrylonitrile and C–H stretching vibrations of benzene in styrene of SAN [38]. In other words, the differences in the FTIR spectra reflect or correspond to the presence of SAN in SAN/PP blends.

### DSC analysis of SAN/PP blends

**Fig. 3:**
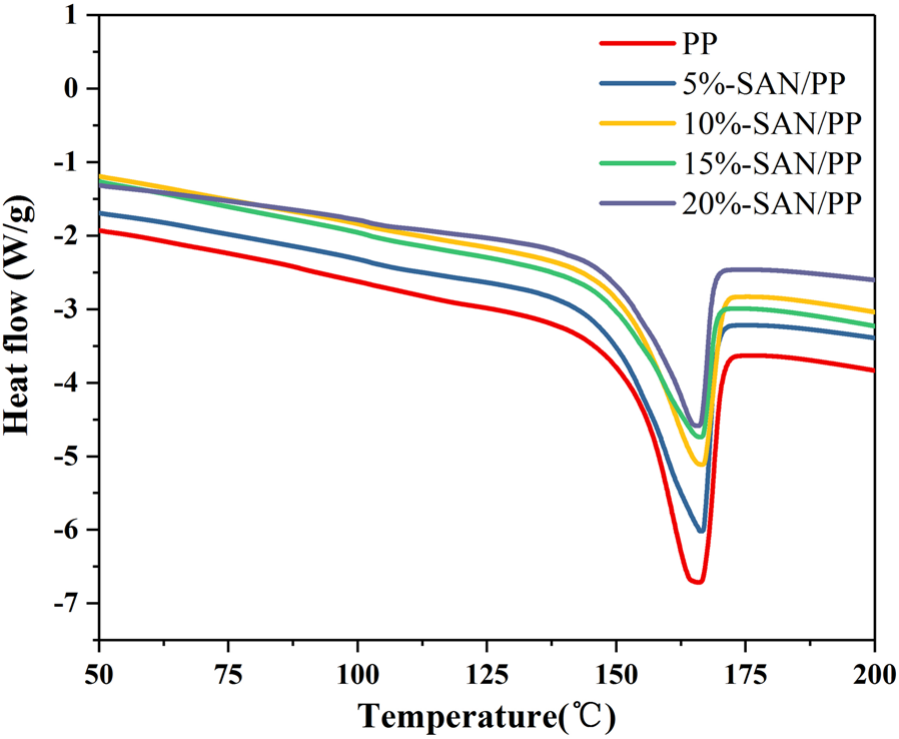
DSC patterns of PP and SAN/PP blends

Melting temperatures (T_m_) of pure PP and SAN/PP blends were examined by DSC. As shown in Fig. 3, the melting point of these specimens were different. The endothermic melting peak occurred at about 165.3 °C for pure PP, which was lower than any of the SAN/PP blends (see Table 2). A similar trend was observed in heat fusion (△H_m_) results, showing that the values of SAN/PP blends containing SAN of 10, 15 and 20 wt% (about 56.6, 48.3, 51.3 J/g respectively) were significantly lower than that of pure PP (about 75.5 J/g) and SAN/PP blends containing SAN of 5 wt% (about 71.0 J/g). In other words, 10 wt% or higher content of SAN in a SAN/PP blend lowers the degree of crystallinity. Only one endothermic melting point was observed, demonstrating that SAN/PP blends crystallized in only one form, and this is consistent with the XRD patterns.

**Table 2:** Melting and crystallization parameters of pure PP and SAN/PP blends

Blends	T_m_ (°C)	△H_m_ (J/g)	X_c_ (%)
SAN/PP-0	166.5	75.5	36.5
SAN/PP-5	167.2	71.0	34.3
SAN/PP-10	167.8	56.6	27.4
SAN/PP-15	168.3	48.3	23.4
SAN/PP-20	167.0	51.3	24.8

These results indicate that SAN has no obviously effect on crystal form but lower the degree of crystallinity of PP. This is different from ABS/PP blends synthesized by several other researchers which obtained β-crystalline phase [16, 17]. Mastan et al. [23] showed that the β crystal form of PP crystals occurrs in HNTs- and IFR-filled 80/20 (wt/wt) PP/ABS blends and their composites, and reasoned that ABS and SEBS-g-MA acted as a β-nucleating agent and the similar depiction by Nayak et al. [16]. However, in our study, SMA and SAN had been added into the PP matrix but did not facilitate the formation of the β-crystal form. This likely correlates with the absence of butadiene for SAN (Fig. 3).

### Scanning electron microscopy

**Fig. 4:**
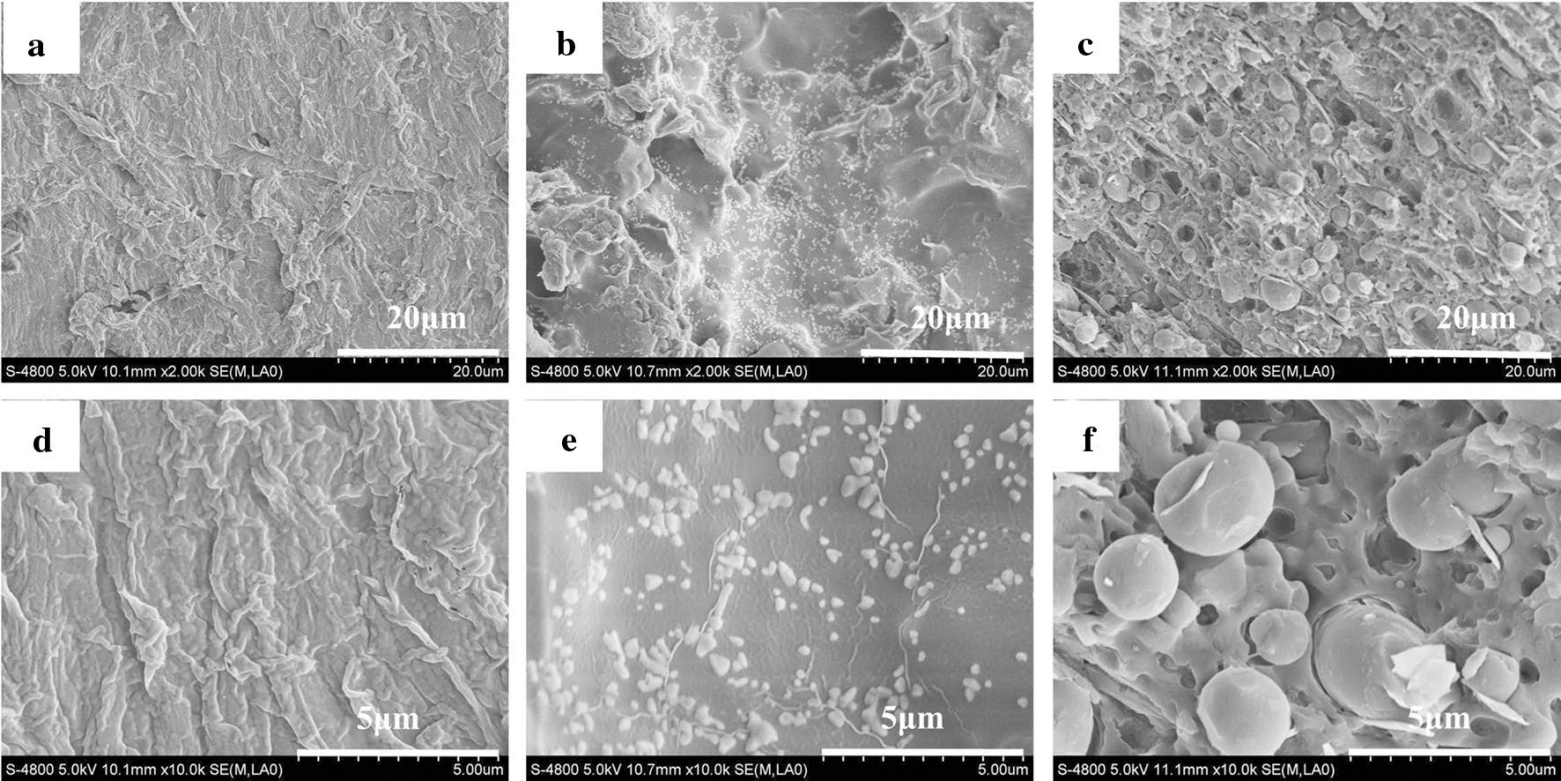
SEM morphologies of the freeze-fractured surface of pure PP 2000 × (**a**) and 10 wt% SAN/PP blends 2000 × (**b**) and 20 wt% SAN/PP 2000 × (**c**) and pure PP 10,000 × (**d**) and 10 wt% SAN/PP blends 10,000 × (**e**) and 20 wt% SAN/PP 10,000 × (**f**)

The morphologies of fracture surfaces of pure PP and SAN/PP blends containing 10 and 20 wt% SAN were investigated by SEM in order to examine the phase compatibility and distribution of SAN in the PP matrix. As shown in Fig. 4, three different kinds of phase morphologies were observed. Some nano-particles with a particle size of 50–900 nm were dispersed on the surface of PP matrix for SAN/PP blends containing 10 wt% (Fig. 4b, e). This was similar with the nanocomposite-doped rigid inorganic filler particles with the fracture morphology of particles distributed on the surface of polymer matrix [39–41]. An irregular structure like sea-island was distinctly observed on surface of SAN/PP blends containing 20 wt% SAN (Fig. 4c). It was mostly covered by SAN spherical droplets and dark holes like meteor crater with the size of 0.5–4 μm (Fig. 4f), indicating the partial miscibility (or intermixing miscibility window) typical of SAN/PP blends [42].

SAN/PP blends containing 10 wt% SAN exhibited the presence of nanoparticles dispersed on the surface, and it was confirmed to be amorphous with no indication of crystal phase by the result of XRD spectrum (Fig. 1). In addition, FTIR spectra (Fig. 2) demonstrated the presence of a C–N band in acrylonitrile and a C–H benzene band in styrene of SAN for SAN/PP blends with 10 wt% SAN. All of these results confirmed that the nanoparticles were mostly correspond to SAN and that was taken as an indication that SAN and PP were utterly immiscible with each other. However, SAN/PP blends containing 20 wt% SAN showed partial miscibility (or intermixing miscibility), between SAN and pure PP, due to unfavorable thermodynamics. And there were many voids on the surface of the specimens, which indicated that the interfacial adhesion of SAN and PP is poor. During the impact fracture, the SAN droplets were pulled out. Some of the spherical droplets which were not pulled out may have arisen from the interaction between the nitrile group of SAN and the maleic anhydride group of SMA [42]. This is consistent with most other researches [43–46]. For example, Kubade and Tambe [36] showed that 80/20 (wt/wt) PP/ABS blend formed coarser matrix-droplet morphology. The result of nanoparticles forming on the continuous surface of SAN/PP blends with 10 wt% SAN is not in agreement with the previous researches related to binary blends. For instance, Krache et al. [42] showed that 10 wt% ABS phase appeared as spherical inclusions in the PC phase matrix. Kim et al. [32] demonstrated that interfacial tension and particle size were further reduced by adding compatibilizer to the PC/SAN blends. Kum et al. [33] obtained the minimum size of the dispersed droplets with an optimized addition compatibilizer ratio on PP/ABS system, which enhanced the interaction between both phases. Thus, in our study, the different morphologies of SAN/PP blends containing 10 wt% SAN and 20 wt% SAN suggested a likely relationship between the size of SAN particles and the compatibility (interaction between SAN and PP).

### Thermal deformation behavior and viscosity analysis

**Fig. 5:**
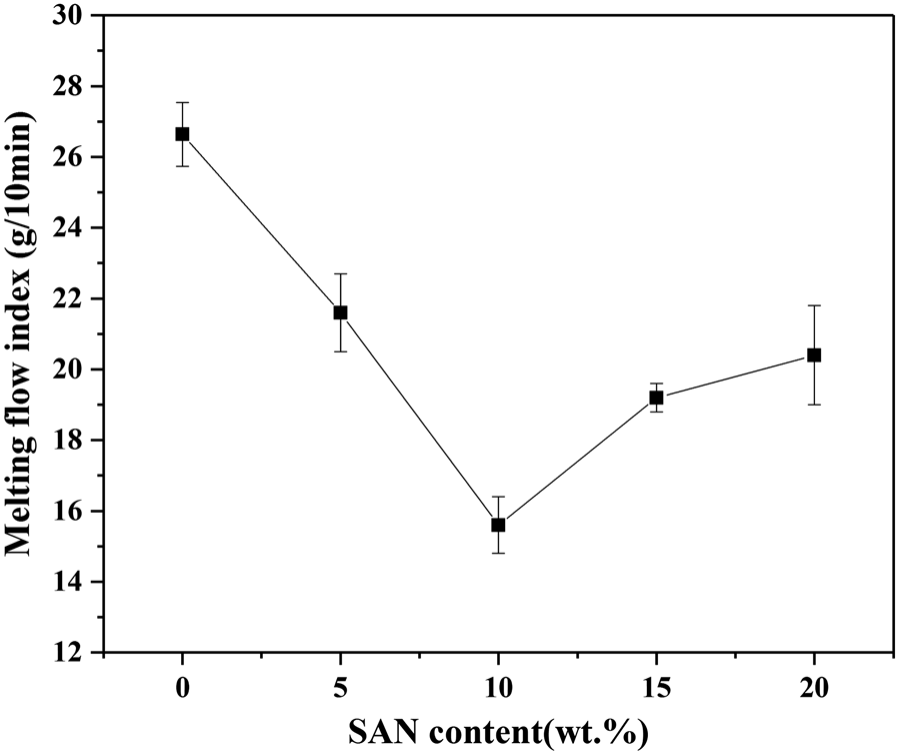
MFI values of PP and SAN/PP blends

It has been reported that the addition of solid particles affects the melting viscosity of polymers [47]. The melt flow indexes of the pure PP and four specimens of SAN/PP blends are shown in Fig. 5. It was found that the curve of MFI values of all specimens appeared as a “V” type. The MFI value reduced sharply as the content of SAN increased, at low concentrations of 10 wt% SAN, followed by an increase observed in the SAN/PP blends with SAN content from 10 to 20 wt%. Overall, the MFI values of all SAN/PP blends were lower than that of pure PP. The similar result was also obtained by other researches with rigid-inorganic/polymer composites [48, 49], in which adding filler particles lowered the MFI. Furthermore, solid particles, such as pigments, fillers or additives, have been reported to affect important rheological properties of polymers, mainly viscosity and deviation from the Newtonian flow [50].

**Fig. 6:**
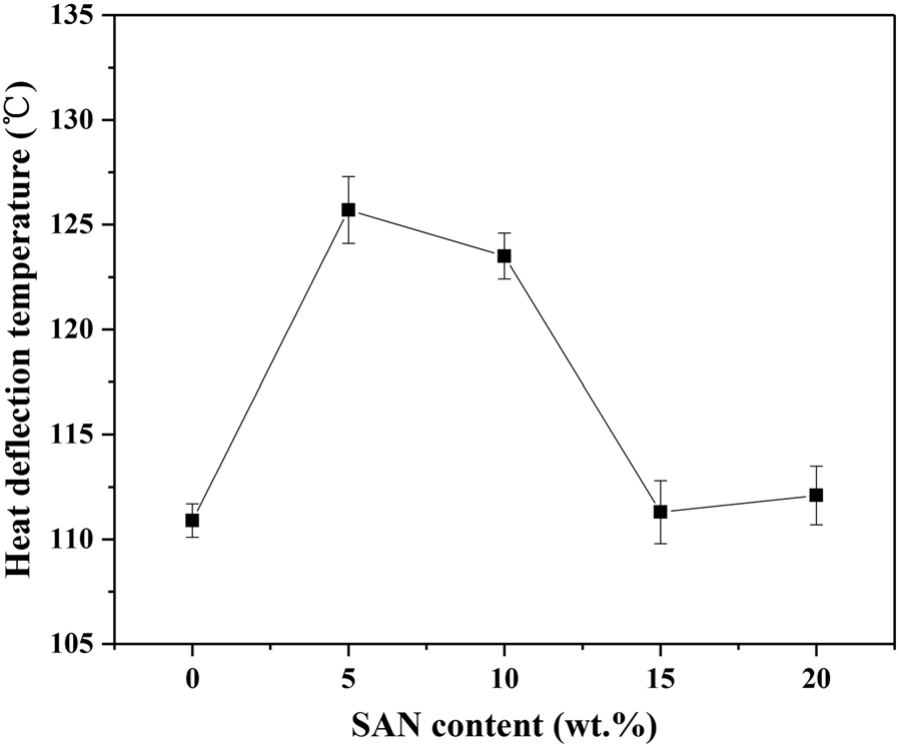
HDT values of PP and SAN/PP blends

**Fig. 7:**
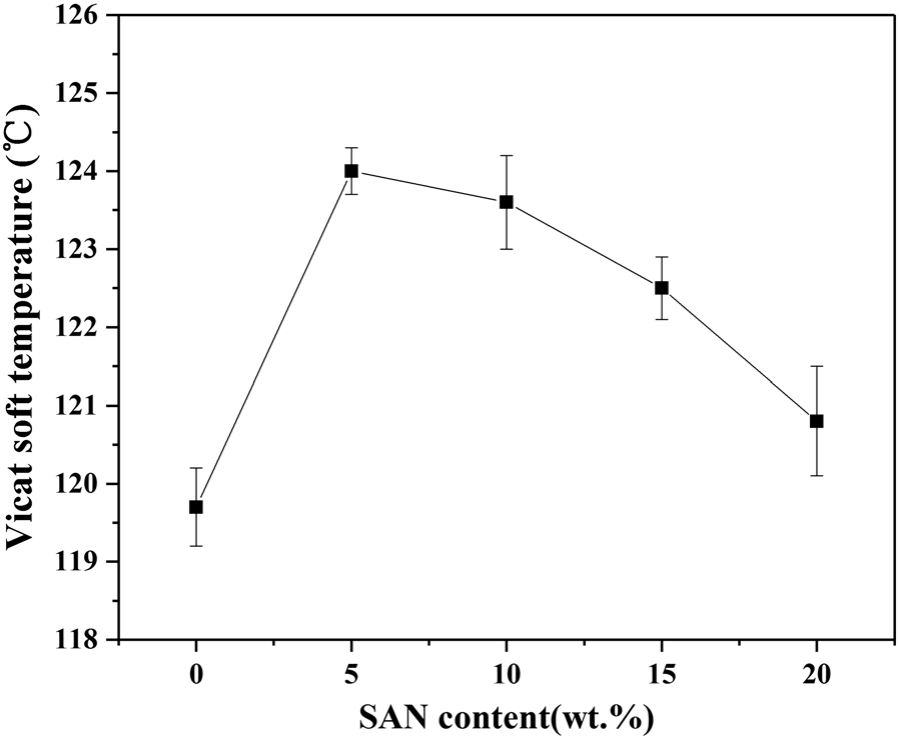
VST values of PP and SAN/PP blends

The Heat Deflection Temperature (HDT) is considered as a function of the temperature of certain creep compliance after the material has been subjected to a certain program [42]. Figures 6 and 7 show the HDT and Vicat Softening Temperature (VST) of pure PP and all SAN/PP blends specimens, respectively. As shown in Fig. 6, the heat deflection temperatures of specimens containing 5 and 10 wt% SAN were distinctly higher than that of pure PP but no obviously elevation was observed for specimens containing 15 to 20 wt% SAN. As for the Vicat points (Fig. 7), the values of all SAN/PP blends were higher than that of pure PP, while decreased as the SAN content increased from 5 to 20 wt%.

**Fig. 8:**
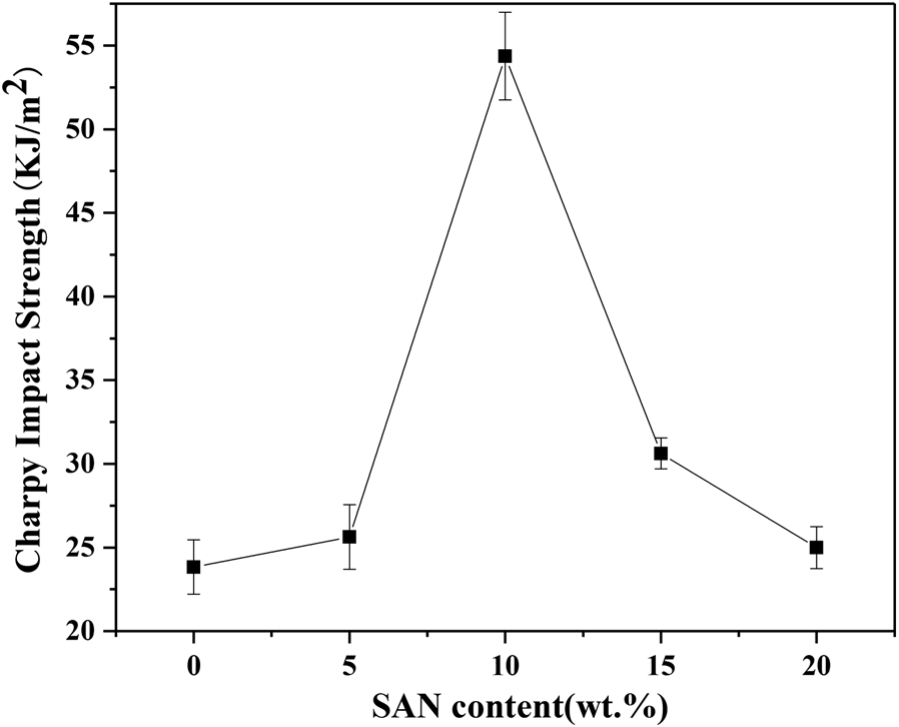
Impact strength of PP and SAN/PP blends

This result suggests that SAN/PP blends exhibit higher HDT and VST values than pure PP, especially for blends with low concentration of SAN (i.e., under 10 wt%). This is not consistent with some other researches, for instance, Krache et al. [42]. showed that the more ABS was added to PC, the lower the HDT and VST values. This difference is likely arising from different phase morphology, in our study, the surface of SAN/PP blends with 10 wt% SAN was covered by rigid nanoparticles. There are some studies, which claims that rigid particle fillers can increase heat distortion temperature of polymers. For example, Qiang and Gubbels et al. [51, 52] demonstrated that rigid fillers improved the heat distortion temperature of polymer blends.

### Mechanical properties

#### Impact strength of SAN/PP blends

**Fig. 9:**
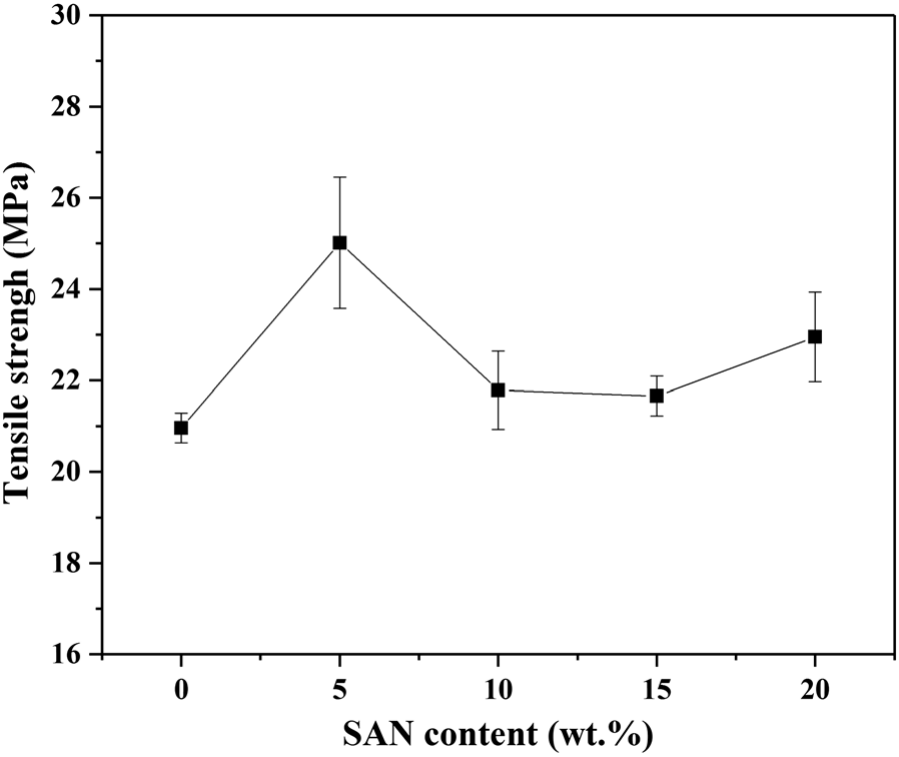
Tensile strength of PP and SAN/PP blends

**Fig. 10:**
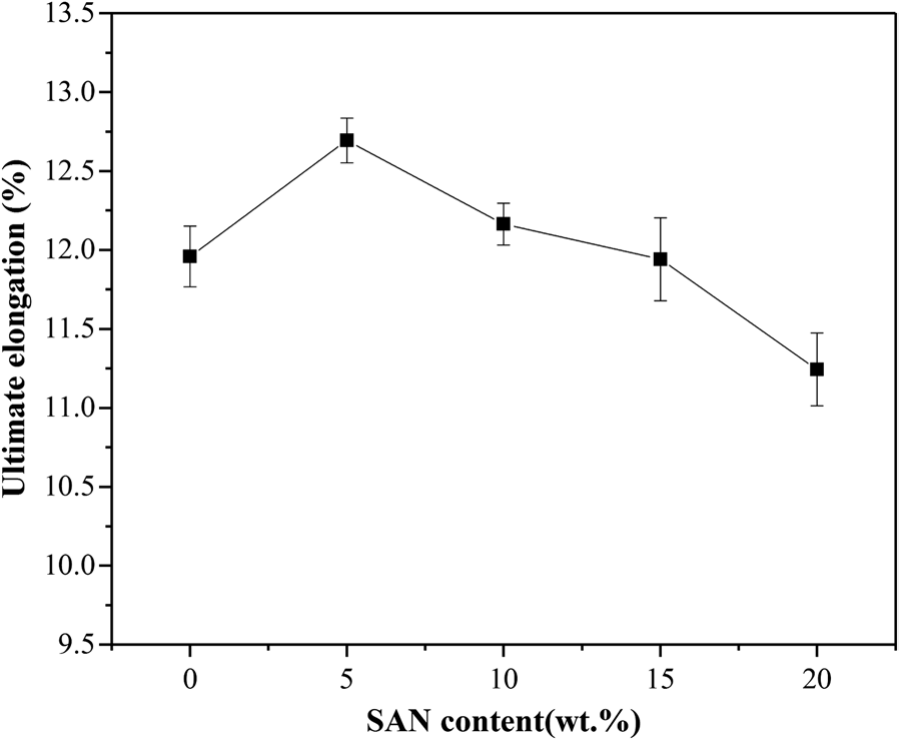
Ultimate elongation of PP and SAN/PP blends

The effects of SAN fillers on the mechanical properties of pure PP are shown in Figs. 8, 9, 10. Figure 8 displays the charpy impact properties of pure PP and SAN/PP blends. Impact strength improved significantly as the amount of SAN increased from 0 to 10 wt%, and then decreased rapidly with the addition of SAN up to 20 wt%. Specifically, the impact strength of SAN/PP blends containing 10 wt% SAN was elevated to 31.59 kJ/m^2^, which was 2.3 times higher than that of pure PP. The impact strength of the other blends containing 5, 15 and 20 wt% SAN showed an increase of 1.79, 6.83 and 1.17 kJ/m^2^, respectively, in contrast to the pure PP. Overall, SAN/PP blends exhibited higher impact strength, especially for blends containing 10 and 15 wt% of SAN.

Impact properties play a critical role in engineering applications. A super-toughened SAN/PP blends with impact strength 2.3 times higher than that of pure PP was achieved by adding 10 wt% SAN. The result reveals that the addition of SAN can significantly improve toughness. This enhancement is likely owing to its phase morphology, with rigid nanoparticles dispersed on the PP surface (Fig. 4) resulting from the incompatibility of SAN and PP. There are some scientific studies, which claim that the addition of rigid particle fillers can increase the impact strength of polymers [28–30]. Sahnoune et al. [53] demonstrated that the incorporation of CaCO_3_ can significantly enhance the stiffness of HDPE/PS blends. Hong et al. [40] showed that the izod impact strength of pure PP is significantly enhanced by adding nano-SiO_2_ particles. García-López et al. [5] claimed that, for a nanocomposite subjected to impact loading, the interfacial regions were able to resist crack propagation more effectively than the polymer matrix. Some researchers have claimed that rigid particle fillers in a polymer matrix under tension would lead to concentrated stress followed by debonding and shear yielding [29]. Besides, the stresses applied to the polymer increase with the increase of the resistance to separation (adhesion strength) between matrix and filler and this resistance is related to particle size. Small particles are desirable when the adhesion between matrix and filler is poor [5]. Although the adhesion needs to be further studied, the particle size in our study is small, and this smallness may have increased resistance to separation as a result of an enhancement of impact properties.

Although the impact strength of SAN/PP blends with 15 and 20 wt% SAN was much lower than that of blends with 10 wt% SAN, when compared to that of pure PP the impact strength of them was slightly improved. This may be attributed to the “sea-island” structure with spherical droplets and dark holes covering the surface of SAN/PP blends. When the impact load was applied to SAN/PP blends, the droplets were pulled out as the load transferring to, followed by void growth at interface or cavitation of SAN, and finally resulted in more energy absorption [23]. On the other hand, it is well known that the mechanical properties of thermoplastics such as tensile, compressive, shear properties and especially impact strength are effected by the degree of crystallinity because the tight molecular arrangement resulting from higher crystallinity will lead to a decline of porosity, restrict the activity of the molecular chain, and ultimately decrease impact strength [54, 55]. Overall, our results showed that SAN/PP blends exhibited higher impact strength than pure PP, but the properties varied according to the amount of SAN. The morphologies of SAN/PP blends with 10 and 20 wt% SAN and and the fact that SAN/PP blends lower crystallinity of PP suggest a close relationship between impact strength, morphology, and crystallinity of SAN/PP blends.

#### Tensile strength of SAN/PP blends

As shown in Figs. 9 and 10, the effects of SAN on the tensile strength and ultimate elongation of blends were examined. It can be seen that the SAN/PP blends containing 5 wt% of SAN exhibited a tensile strength of 25.0 MPa, which was higher than that of pure PP (20% over than pure PP), and had an higher elongation of 12.7%. As the SAN concentration increased, the tensile strength was slightly higher than that of pure PP. When the concentration increased up to 20 wt%, the elongation was reduced to 11.24%. Generally, 5 wt% of SAN in SAN/PP blends showed a maximum values of tensile strength and ultimate elongation, which was attributed to the refined dispersion of nanoparticles in PP matrix [56].

## Conclusion

In summary, we demonstrated that SAN/PP blends with different content of SAN showed different morphologies, mechanical performances and thermal deformation properties. According to the XRD, FTIR and DSC analyses, SAN had no obviously effect on crystal form but reduced the crystallinity of PP. Thermal deformation and viscosity assays showed that the addition of SAN to PP increased the viscosity of blends and HDT and VST values were enhanced for all SAN/PP blends. The SAN/PP blends with 10 wt% SAN revealed the presence of nanoparticles dispersed on the surface, while SAN/PP blends with 20 wt% SAN exhibited sea-island morphology. All SAN/PP blends showed higher impact strength compared to pure PP, especially for SAN/PP blend containing 10 wt% SAN. The reason for the significant increase was most likely related to formation of rigid nanoparticles and the slight increase for SAN/PP blends with 15 and 20 wt% SAN was likely owing to the sea-island morphology and the decrease of crystallinity.
